# Complex Genetic Framework in Familial Amyotrophic Lateral Sclerosis With a C9ORF72 Mutation: A Case Report

**DOI:** 10.7759/cureus.76027

**Published:** 2024-12-19

**Authors:** Andrey Frolov, Elizabeth D'sa, Camille Henderson, Miguel A Guzman, Ghazala Hayat, John R Martin

**Affiliations:** 1 Department of Surgery - Center for Anatomical Science and Education, Saint Louis University School of Medicine, St. Louis, USA; 2 Department of Pathology, Saint Louis University School of Medicine, St. Louis, USA; 3 Department of Neurology, Saint Louis University School of Medicine, St. Louis, USA

**Keywords:** cancer, familial amyotrophic lateral sclerosis, neuromuscular diseases, next generation sequencing (ngs), whole exome sequencing (wes)

## Abstract

A significantly diverse clinical presentation of amyotrophic lateral sclerosis (ALS), even in its best-studied familial form, continues to hinder current efforts to develop effective disease-modifying drugs for the cure of this rapidly progressive, fatal neuromuscular disease. We have previously shown that clinical heterogeneity of sporadic ALS (sALS) could be explained, at least in part, by its polygenic nature as well as by the presence of mutated genes linked to non-ALS neurological diseases and genes known to mediate ALS-related pathologies. We hypothesized that a similar genetic framework could also be present in patients with familial ALS (fALS). To test this hypothesis, we conducted post-mortem genetic screening of an individual with fALS and a mutation in the* C9ORF72 *gene. *C9ORF72* mutations are highly penetrant and are present in the majority of fALS patients. Genetic screening by whole exome sequencing (WES) on the next generation sequencing (NGS) Illumina platform (San Diego, CA, USA) followed by examination of the respective rare (minor allele frequency (MAF) ≤ 0.01) pathological/deleterious genetic variants yielded results consistent with our hypothesis of the presence of a complex genetic framework in fALS. Additional members of this genetic framework were identified when the low-frequency (0.01 < MAF < 0.05) pathological/deleterious genetic variants were analyzed with the low-frequency biallelic *AHNAK2,* *GLI3, PTIRM1, *and* ZNF254 *variants, warranting a closer look at their potentially important role in fALS as *C9ORF72* genetic modifiers as well as their link to both neuromuscular disorders/ALS and cancer. Therefore, in addition to the current genetic screening using a standard panel of ALS-related genes, a supplementary screening by WES could be very beneficial for the development of personalized treatment of ALS patients as well as in search of the respective efficient disease-modifying drugs.

## Introduction

Amyotrophic lateral sclerosis (ALS) is a progressive fatal neurodegenerative disease affecting one to three individuals per 100,000 people annually worldwide [1]. The ALS occurrence was predicted to increase by around 69% from 2015 to 2040 mostly because of population aging [2]. ALS is an aggressive disorder characterized by a loss of motor neurons which is accompanied by muscle atrophy and weakness affecting limb and cranial muscles, eventually leading to death within two to four years after the disease onset primarily due to the failure of respiratory muscles [3]. One of the distinct ALS features is its remarkably diverse clinical presentation which is not only observed in patients with different causative mutations [4] but also in patients with the same affected gene. The latter could be best illustrated by the familial form of ALS (fALS) with patients bearing a drastically increased number of GGGGCC hexanucleotide tandem repeat expansions in the first intron of the *C9ORF72* gene [5]. Such genetic alteration accounts for 30-50% of all fALS cases [6] and *C9ORF72* autosomal dominant mutations produce three distinct clinical phenotypes: ALS alone, frontotemporal dementia (FTD) alone, or the combination of both (ALS-FTD) [5-7]. The remarkable ALS clinical heterogeneity poses a significant challenge not only for clinicians diagnosing ALS and identifying protocols for the most efficient and personalized treatment of ALS patients, but also for researchers developing effective disease-modifying drugs. Therefore, advancing our understanding of the molecular basis of ALS clinical heterogeneity could improve treatment and care of ALS patients as well as optimize a strategy for the development of efficient disease-modifying drugs.

We have previously probed the molecular basis of ALS clinical heterogeneity in two patients with sporadic ALS (sALS) by performing post-mortem genetic screening using DNA whole exome sequencing (WES) on the Illumina next generation sequencing (NGS) platform (San Diego, CA, USA) [8]. The sporadic form of ALS accounts for approximately 85% of all ALS cases and is characterized by a highly heterogeneous clinical phenotype [9]. Our previous results were consistent with the existence in sALS of a complex framework of mutated genes including those directly linked to ALS, genes associated with non-ALS neurological disorders, and genes involved in ALS-related pathology [8]. Given the remarkable clinical heterogeneity of fALS as well as its significant genetic and phenotypical overlap with non-ALS neurological disorders [4,6,7], we hypothesized that a genetic framework similar to that of sALS could also exist in fALS. In the current report we provide evidence in support of our hypothesis.

## Case presentation

Body donor

The body of a 56-year-old female was received through the Saint Louis University (SLU) Gift Body Program with signed informed consent.

Donor medical history

A 55-year-old female presented with progressive weakness and fasciculations in her lower and upper extremities. Two years earlier she began having muscle twitching and weakness in her lower extremities that progressed to affect her arms. She denied experiencing dysphagia or dysarthria. Her family history was significant as her grandmother was diagnosed with ALS and both parents had unspecified cancer. Past medical history was significant for hypertension, pre-diabetes mellitus, vitamin B12 deficiency, and alcohol use disorder. Neurological examination showed normal mental status. Cranial nerve examination was normal and there was no evidence of tongue fasciculations. Fasciculations were noted on examination of the triceps and quadriceps muscles. Bilateral upper extremities proximal strength was 3+/5. Bilateral lower extremities distal strength was 3/5. Reflexes were 3+ with absent bilateral ankle jerks. A magnetic resonance imaging (MRI) scan of the brain was unremarkable whereas MRI scans of the cervical, thoracic and lumbar spine demonstrated degenerative disc disease. Creatine kinase levels were elevated to the 500s U/L values. An antinuclear antibody (ANA) test was negative. Electrodiagnosis showed denervation in four body regions: cranial, cervical, thoracic and lumbar. The genetic testing was conducted by the PreventionGenetics Laboratory (Marshfield, WI, USA) and was positive for *C9ORF72* mutation. The donor died a year later, four years after her initial symptoms began.

In summary, the medical history of the donor describes a common case of fALS with a highly penetrant *C9ORF72* causative mutation and without dementia. For the genetics studies described below, only key case identifiers such as its familial type, *C9ORF72* mutation, and unspecified cancer in both parents will be used for the data analysis and discussion.

Genetic analysis

The post-mortem genetic screening by WES on Illumina NGS platform and the respective bioinformatics analysis were performed as previously described [8]. The cumulative exome coverage for 50x depth of coverage was 93%. Gene grouping was based on the GeneCards, Google Scholar, and PubMed searches.

Results

The genetic screening of the donor yielded 78 genes with rare (minor allele frequency (MAF) ≤ 0.01) pathological /deleterious variants that were grouped into the following categories: genes linked to ALS (Group I, Table 1), genes linked to non-ALS neurological diseases (Group II, Table 2), and genes linked to biological processes perturbed in ALS (Group III, Table 3).

**Table 1:** Genes with rare pathological/deleterious variants in the donor linked to amyotrophic lateral sclerosis (ALS). *Model system evidence.

Categories		Genes
Causative, risk, or functionally related genes		*FMN1* [10], *HLA-DOA* [10], *HPF1* [11], *IFRD1* [12], *NOD2* [13], *SLC17A3* [14], *TTN* [15]
Differentially regulated genes	Expression	*CLIC5* [16]*, *FMN1 *[17], *GATA5* [18], *HLA-DOA* [19],* IFRD1 *[20]*, *MICALL2* [21]*, *MYL3 *[22, 23], *NOD2* [24], PARD3B*, *PTPRG *[25]*, *SIRT2* [26], *SLC26A4* [17]
	Epigenetic regulation	*ATAD3B* [27]
Genes encoding differentially regulated proteins	Protein expression	*CRTAC1* [28, 29], *LTA4H* [30], *MEGF8* [28], *MYL3* [31], *NOD2* [24], *SIRT2* [26], *TTN* [32]
	Protein activity	*ACO2* [33]

**Table 2:** Genes with rare pathological/deleterious variants in the donor linked to non-amyotrophic lateral sclerosis (ALS) neurological diseases. * Model system evidence.

Disease/Disorder Type	Genes
Neuromuscular and Neurodegenerative	ATG7, CPZ, CRTAC1, DGKG, HPF1, IFNA14, IFRD1, IGFLR1, LRP8*, LTA4H, MOCS2, MRPL2, MYH13, NUDT22, TBL3, TESPA1, TTN, USP13, ZRANB3*
Neurodevelopmental	*AAF2, ATAD3B, CLIC5, CLTCL1, GAL3ST2, GCFC2, GRIN3B, ICE1, ITSN1, MEGF8, MICALL2, MOCS2, MRPL2, MYH13, NAXD, OR11H12, PIWIL4, PTPRG, TBC1D10A, TESPA1, USP13*
Neuropsychiatric	GRIN3B, LRP8, PPP6R1, SLC17A3
Alzheimer’s	MEGF8, MSR1, OR11H12, PTPRG, USP13
Parkinson’s	ACO2, FCHO1, GATA5, HPF1, ICE1, MEGF8, MSR1, OR11H12, SIRT2, USP13
Huntington’s	RGS9
Stroke	FN3KRP

**Table 3:** Genes with rare pathological/deleterious variants in the donor linked to biological processes perturbed in amyotrophic lateral sclerosis (ALS).

Biological Process	Genes
Neuronal differentiation and function	ACBD6, ATG7, CLTCL1, EXOC1, PARD3, RGS9, SEMA6C, SIRT2, TBC1D10A, TESPA1, TLN2, TRPPV3, MICALL2
Vesicular transport	CLTCL1, EXOC1, TBC1D10A, MICALL2
Nuclear transport	EIF4ENIF1
Intracellular signaling	DGKG, PARD3, RGS9, SEMA6C, TESPA1, TRPV3
Mitochondrial function	ACO2, ATAD3B
Autophagy	ACO2, ATG7, EXOC1
Expression and toxicity of alleles with expanded repeats	AFF2, EXOC1
Epigenetic gene regulation	SIRT2
Lipid metabolism	ATAD3B, DGKG, MSR1
Skeletal muscle function	MOCS2, MYH13, SEMA6C
Immunity and inflammation	MRGPRX2, MSR1, SIRT2, TESPA1

Interestingly, despite the presence of highly penetrant *C9ORF72* mutation in the donor, several other mutated genes classified in the literature as ALS causative, risk, or functionally related to ALS were also identified (Table 1). Table 2 lists multiple entries for genes linked to non-ALS neuromuscular and neurodevelopmental diseases including those of major neurological diseases such as Alzheimer’, Parkinson’s, and Huntington’s diseases. The affected genes listed in Table 3 were linked to many important biological processes known to be perturbed in ALS [34-36].

The analysis of low-frequency mutations (0.01 < MAF < 0.05) yielded 68 genes with pathological/deleterious variants including additional multiple entries for Groups I-III (Tables 4-6). One of the notable features of this dataset was the presence of biallelic genetic variants of *AHNAK2* (Tables 5, 6), *GLI3* (Tables 4, 6), *PITRM1* (Tables 5, 6), and *ZNF254* (Tables 4, 6).

**Table 4:** Genes with low-frequency pathological/deleterious variants in the donor linked to amyotrophic lateral sclerosis (ALS). * Model system evidence.

Categories		Genes
Causative, risk, or functionally related genes		*AKR1C3* [37], *MRPS27* [38], *SGCD* (GeneCards), *TM6SF1* (GeneCards), *TTN *[15]
Differentially regulated genes	Expression	*ATP10B* [17], *CCDC114* [17], *COL18A1* [39], *CPT2* [40]*, *GAS2L3* [41]*, *LARP4* [42]*, *NEUROD1* [43], *SGK1 *[44]*, *ZNF254* [45]*
	Epigenetic regulation	*FAM149A* [46]
Genes encoding differentially regulated proteins	Protein expression	*ABHD14A* [47]*, *ACAA1* [48], *GLI3* [49]*, *TTN* [32]

**Table 5:** Genes with low-frequency pathological/deleterious variants in the donor linked to non-amyotrophic lateral sclerosis (ALS) neurological diseases. * Model system evidence.

Disease/Disorder Type	Genes
Neuromuscular and Neurodegenerative	ABHD14A, ACADS, AZIN2, CPT2, GAS2L3, INSC, MYBC2, OR2B2, PITRM1, SAMD9L, SGCD, SPATA5L1, TFDP3, TMEM176B, TRIM22, TRIM49, TTC22, TTN, TTC22
Neurodevelopmental	ABHD14A, AHNAK2, ATP10B, BTN2A1,COL18A1, DAGLB, GPR37, KRT16, NEUROD1, NMBR, OR2B2, PUS3, TM6SF1, TNRC18, TTC22
Neuropsychiatric	BTN2A1, CCDC107
Alzheimer’s	AZIN2, BTN2A1, CLNK, FBXW12, PITRM1
Parkinson’s	ATP10B, GPR37, SGK1*
Huntington’s	MPHOSPH8
Charcot-Marie-Tooth	AHNAK2, CLNK
Bardet-Biedl Syndrome	SDHAF3

**Table 6:** Genes with low-frequency pathological/deleterious variants in the donor linked to biological processes perturbed in amyotrophic lateral sclerosis (ALS).

Biological Process	Genes
Neuronal differentiation and function	COL18A1, KRT16
Gene Transcription	NEUROD1, TFDP3, ZNF254, ZNF44
Vesicular transport	CCDC114
DNA repair	MUS81
Intracellular signaling	AHNAK2, GLI3, TBC1D32
Mitochondrial function	ACAA1, ACADS, CPT2, SDHAF3
Protein translation	PUS3
Protein degradation	PITRM1
Apoptosis	BCL2L14
Cell stress response	SGK1
Lipid metabolism	ACAA1, ACADS, CPT2, DAGLB
Immunity and inflammation	AKR1C3, SAMD9L, TNIP2, TRIM22

## Discussion

The genetic screening by WES of the individual with fALS and *C9ORF72* highly penetrant mutation provided two very important lines of information. The first important set of evidence was pertinent to our understanding of the molecular basis of ALS clinical heterogeneity. Indeed, the data obtained supports our hypothesis regarding the presence in fALS of the genetic framework similar to that of sALS. This genetic framework was mainly formed by three groups of genes including those directly linked to ALS (Group I), genes linked to non-ALS neurological diseases (Group II), and genes associated with major biological processes known to be perturbed in ALS (Group III). Therefore, one could hypothesize that an individual with fALS could be primed for the disease by the Group III mutated genes. ALS could be then triggered not solely by a powerful mutation in a single gene such as *C9ORF72*, but would rather commence through a concerted action of the latter and the affected genes from Group I. Once triggered, the respective fALS clinical phenotype will be formed in a manner similar to that suggested for sALS [8] by the mutated genes from Groups II and III. Importantly, the number and type of genes in the fALS genetic framework could vary between individuals which could explain the diverse fALS clinical presentation even in the setting of the same causative mutation [5-7].

The second important set of evidence obtained during this study was related to crosstalk between neurodegenerative diseases (NDD), including ALS, and cancer where the mechanisms regulating NDD and cancer share some of their respective components [50]. A structural and functional link between ALS and cancer was further supported by reports where most of all 101 potential ALS biomarkers were associated with various cancer types [51] and where antecedent ALS or cancer affected each other’s development risks [52]. The apparent link between ALS and cancer was also evidenced by a successful application of a cancer multistep model to describe the incidence of ALS in humans [53-55]. With this regard, the presence of four low-frequency biallelic pathological/deleterious genetic variants of *AHNAK2, GLI3, PITRM1,* and *ZNF254* was intriguing. Indeed, besides their potentially high impact on the respective clinical phenotype in the autosomal recessive manner, each one of them is linked to both neuromuscular disorders/ALS and cancer. *AHNAK2* is a causative gene in autosomal recessive form of Charcot-Marie-Tooth disease (CMT) [56] which has significant genetic and clinical symptoms overlap with that of ALS [57]. *AHNAK2* is also an oncogenic protein acting through the TGF-β/Smad3 signaling pathway [58]. *GLI3* functions in the Sonic Hedgehog pathway which is neuroprotective in a model ALS [59] and Gli3 protein expression is reduced in motor neurons of a mouse ALS model [49]. Yet GLI3 along with androgen receptor displays oncogenic activity in vitro [60]. *PITRM1* biallelic mutations have been linked to autosomal recessive spinocerebellar ataxia [61] and may participate in cancer cell survival mediated by linear noncoding RNA *SNHG5* [62]. Finally, *ZNF254* regulates development of motor neurons and could be one of the affected genes in ALS [45]. It may also have a role in oncogenesis [63] and serve as a potential peptide biomarker for ovarian cancer [64]. Therefore, it would be very interesting to study a large cohort of fALS patients for the presence of similar pleiotropic low-frequency biallelic genetic variants. 

## Conclusions

The results of the present work provide a plausible explanation for the fALS clinical heterogeneity and suggest an addition of the genetic screening by WES to the genetic testing of patients with the standard panel of ALS-related genes. Such additional genetic testing could significantly advance the diagnosis and personalized treatment of patients with ALS as well as the development of efficient disease-modifying drugs. The presence in the current case of low-frequency biallelic genetic variants linked to both ALS and cancer could provide important insights into the molecular mechanism(s) underlining both diseases.

Despite providing important evidence in support of our hypothesis, the fact that it was based only on one individual and the studies were conducted postmortem, the current report can only be viewed as a starting point for the follow-up antemortem studies employing a large cohort of fALS patients and/or using the respective clinical databases.
